# Impairment of cardiac metabolism and sympathetic innervation after aneurysmal subarachnoid hemorrhage: a nuclear medicine imaging study

**DOI:** 10.1186/cc13943

**Published:** 2014-06-25

**Authors:** Bertrand Prunet, Mathieu Basely, Erwan D’Aranda, Pierre Cambefort, Frédéric Pons, Sébastien Cimarelli, Arnaud Dagain, Nicolas Desse, Jean-Brice Veyrieres, Christophe Jego, Guillaume Lacroix, Pierre Esnault, Henry Boret, Philippe Goutorbe, Emmanuel Bussy, Gilbert Habib, Eric Meaudre

**Affiliations:** 1 Department of Critical Care, Service de Réanimation, Sainte Anne Military Teaching Hospital, Boulevard Sainte Anne, Toulon 83000, France; 2 Department of Nuclear Medicine, Sainte Anne Military Teaching Hospital, Boulevard Sainte Anne, Toulon 83000, France; 3 Department of Cardiology, Sainte Anne Military Teaching Hospital, Boulevard Sainte Anne, Toulon 83000, France; 4 Department of Nuclear Medicine, Léon Bérard Cancer Institute, 28, rue Laennec, Lyon 69373, France; 5 Department of Neurosurgery, Sainte Anne Military Teaching Hospital, Boulevard Sainte Anne, Toulon 83000, France; 6 Department of Neuroradiology, Sainte Anne Military Teaching Hospital, Boulevard Sainte Anne, Toulon 83000, France; 7 Department of Cardiology, Timone Hospital and Aix-Marseille University, 27, boulevard Jean Moulin, Marseille 13385, France

## Abstract

**Introduction:**

Although aneurysmal subarachnoid hemorrhage (SAH) is often complicated by myocardial injury, whether this neurogenic cardiomyopathy is associated with the modification of cardiac metabolism is unknown. This study sought to explore, by positron emission tomography/computed tomography, the presence of altered cardiac glucose metabolism after SAH.

**Methods:**

During a 16-month period, 30 SAH acute phase patients underwent myocardial ^18^ F- fluorodesoxyglucose positron emission tomography (^18^F-FDGPET), ^99m^Tc-tetrofosmin and ^123^I-meta-iodobenzylguanidine (^123^I-mIBG) scintigraphy, respectively, assessing glucose metabolism, cardiac perfusion, and sympathetic innervation. Patients with initial abnormalities were followed monthly for two months for ^18^F-FDG, and six months later for ^123^I-mIBG.

**Results:**

In this SAH population, acute cardiac metabolic disturbance was observed in 83% of patients (n = 25), and sympathetic innervation disturbance affected 90% (n = 27). Myocardial perfusion was normal for all patients. The topography and extent of metabolic defects and innervation abnormalities largely overlapped. Follow-up showed rapid improvement of glucose metabolism in one or two months. Normalization of sympathetic innervation was slower; only 27% of patients (n = 8) exhibited normal ^123^I-mIBG scintigraphy after six months. Presence of initial altered cardiac metabolism was not associated with more unfavorable cardiac or neurological outcomes.

**Conclusions:**

These findings support the hypothesis of neurogenic myocardial stunning after SAH. In hemodynamically stable acute phase SAH patients, cardiomyopathy is characterized by diffuse and heterogeneous ^18^F-FDG and ^123^I-mIBG uptake defect.

**Trial registration:**

Clinicaltrials.gov NCT01218191. Registered 6 October 2010.

## Introduction

Subarachnoid hemorrhage (SAH) following aneurysm rupture remains a devastating condition with high mortality and poor outcome among survivors [1,2]. Recent developments in neurocritical care have reduced the mortality rate from 50% to 25 to 35% [3]. Medical non-neurological complications add to morbidity and mortality, rivaling the frequency of mortality from neurological complications [3-5]. Myocardial abnormalities have been reported in 50 to 100% of patients with severe SAH [6], and may include electrocardiogram (ECG) changes [7], troponin Ic elevation with myocardial necrosis [6,8], increased B-type natriuretic peptide (BNP) level [9], and cardiogenic shock. Despite controversies, the neurogenic hypothesis is now the most commonly held theory of pathogenesis of this acute stress cardiomyopathy [10-12].

We hypothesize that cardiac metabolism is modified after SAH. The primary objective of this study was to observe the impairment of cardiac glucose metabolism and to quantify its incidence and reversibility in a population of SAH patients. In addition, the duration and reversibility of cardiac neurogenic sympathetic injury after SAH remain unknown, and were evaluated as a secondary objective during the acute period and six months later.

## Materials and methods

### Patients

From November 2010 through February 2012, we recruited adults with aneurysmal SAH in the intensive care unit (ICU) of the Sainte Anne Military Teaching Hospital, Toulon, France. Eligibility criteria for inclusion were the following: SAH related to a ruptured aneurysm documented by angiography, and age over 18 years. Patients, families, or referring physicians were interviewed to determine the date and nature of the first clear signs or symptoms of SAH. If the delay from the first sign or symptom of aneurysm rupture to arrival at the ICU was more than 48 h, patients were not included. Additional exclusion criteria were pregnancy, past medical history of ischemic heart disease or chronic heart failure, and insufficient stability to allow intrahospital transport to the Nuclear Medicine Department (patients on vasopressor or inotrope, arterial partial pressure of oxygen/fractional inspired oxygen ratio under 200, fractional inspired oxygen over 60%, intracranial pressure over 20 mmHg). Patients who died before the first isotopic procedure were excluded. The study protocol was approved by a national ethics review board for human subjects (Comité pour la Protection des Personnes Sud Méditerranée V, Nice, France). In all cases, the patients’ next of kin provided written informed consent.

### Study procedures

All patients were admitted to our unit for at least a seven-day period, and were managed according to the French Society of Anesthesiology and Intensive Care guidelines [13].

Clinical and demographic data were collected. Each patient’s neurological status was assessed at the time of admission and graded according to the World Federation of Neurosurgical Societies (WFNS) and the scanographic Fisher’s scale. Data regarding aneurysmal treatment and neurological events were also recorded.

Vasospasm was detected by clinical evaluation and daily transcranial Doppler, and then diagnosed by cerebral angiography. Vasospasm was managed by hypertension and hemodilution, and intracranial angioplasty when possible. No patient enrolled in this study required vasopressor medication during the 24 h prior to isotopic examination.

Delayed cerebral ischemia was defined as development of focal neurologic signs or deterioration of the level of consciousness, or both, with evidence of cerebral infarction on CT scan, or any new hypodensity on the CT scan without an obvious explanation such as neurosurgical or endovascular intervention, or perihematomal edema even in the absence of clinical symptoms.

#### Scintigraphic procedures

All isotopic procedures were performed in the Nuclear Medicine Department of the Sainte Anne Military Teaching Hospital and interpreted by two of three experienced nuclear medicine physicians (MB, PC, and EB) blinded to the clinical status of the patients. If necessary, a consensus reading was made.

### Myocardial glucose metabolism

Myocardial glucose metabolism was assessed by cardiac ^18^F-fluorodesoxyglucose positron emission tomography (^18^F-FDG PET), which was performed as soon as possible after stability was achieved. When an initial abnormality was observed, a second examination was performed one month later. When an abnormality persisted at the one-month examination, the examination was repeated again one month later. PET was performed in accordance with the 2003 American Society of Nuclear Cardiology Practice Guidelines on PET myocardial glucose metabolism imaging [14]. Each patient fasted for 6 h, and then was administered a standardized oral glucose load of 90 g. The targeted blood glucose level was 100 to 150 mg/dl obtained, if needed, with insulin infusion according to guidelines [14]. ^18^F-FDG intravenous injection was performed approximately 1 h after glucose loading, with a PET acquisition began 45 minutes after the 185 MBq ^18^ F-FDG using combined PET/computed tomography (CT) technology (Siemens Biograph BGO, Siemens Healthcare, Erlangen, Germany). A thoracic CT scan was performed just before a 15-minute three-dimensional PET acquisition. PET data were reconstructed with and without CT-based attenuation correction using an iterative technique. PET reconstructed images were realigned along the short axis and the vertical and horizontal long axes and qualitatively interpreted. A 17-segment model of polar map presentation was obtained from left ventricular (LV) short-axis slices. PET images were interpreted using QPS-QGS software (Cedars-Sinai, Los Angeles, CA, USA), and myocardial uptake defects were quantified as a percentage of the entire LV wall. Myocardial glucose metabolism was considered abnormal if the ^18^F-FDG uptake defect area was greater than an upper threshold value of 15%.

### Myocardial sympathetic innervation

Myocardial sympathetic innervation was assessed using cardiac ^123^I-meta-iodobenzylguanidine (^123^I-mIBG) scintigraphy, which was performed as soon as possible after stability was achieved. When an initial abnormality was observed, a second examination was performed six months later. ^123^I-mIBG scintigraphy was performed in accordance with the European Association of Nuclear Medicine Guidelines [15]. ^123^I-mIBG is an analog of noradrenaline; decreased myocardial uptake of ^123^I-mIBG indicates sympathetic nerve dysfunction [15]. When it was possible, medical therapy and drugs known to influence ^123^I-mIBG uptake were discontinued for at least 24 h before tracer injection [15]. Thereby, according to the 2010 European Association of Nuclear Medicine guidelines [15], administration of nimodipine to prevent vasospasm was discontinued for 24 h before this examination. Thyroid uptake of ^123^I was prevented with the oral administration of 130 mg of potassium iodide one day before and after the planned ^123^I-mIBG scintigraphy. Four hours after the intravenous injection of 220 MBq ^123^I-mIBG, cardiac ^123^I-mIBG scintigraphy was performed using a double-headed gamma camera (Siemens Symbia E, Siemens Healthcare, Erlangen, Germany) equipped with low-energy, high-resolution, parallel-hole collimators. We acquired a 10-minute planar imaging series in the anterior position from a 64 × 64 matrix, as well as a single-photon emission computed tomography (SPECT) series with 32 60-s projections (180°, 64 × 64 matrix). The SPECT series was reconstructed using ordered-subsets expectation maximization iterative technique without attenuation or scatter correction, and realigned along the heart axis. To quantify ^123^I-mIBG uptake, heart to mediastinal (H/M) average count ratio was used on the planar acquisitions. The heart region of interest (ROI) was drawn manually to include both ventricles and any clearly visible atrial activity. A square mediastinal ROI was drawn in the upper mediastinum, using the apices of the lungs as anatomic landmarks. The H/M ratio was calculated as the ratio of the counts/pixel in the two ROIs [16]. Myocardial sympathetic innervation was considered normal if the H/M ratio exceeded a recommended threshold value of 1.75 [17]. A 17-segment model of polar map presentation was obtained from LV short-axis slices. Regions of low or absent ^123^I-mIBG uptake indicated myocardial sympathetic impairment. For quantitative analysis of radionuclide uptake, myocardial uptake defects were quantified as a percentage of the entire LV wall using QPS-QGS software (Cedars-Sinai, Los Angeles, CA, USA).

#### Myocardial perfusion

Rest myocardial perfusion was assessed by cardiac ^99m^Tc-tetrofosmin gated single photon emission computed tomography (G-SPECT) scintigraphy, which was performed as soon as possible after stability was achieved. Since the simultaneous use of radiotracers could result in an important complicating cross-talk of energy spectra, ^99m^Tc-tetrofosmin G-SPECT scintigraphy was often performed the preceding day of the ^123^I-mIBG scintigraphy. When an initial abnormality was observed, a second examination was performed six months later. G-SPECT was initiated 20 minute after ^99^mTc-tetrofosmin intravenous injection (740 MBq) using a double-headed gamma camera (Siemens Symbia E, Siemens Healthcare, Erlangen, Germany) equipped with low-energy, high-resolution, parallel-hole collimators; a 180° rotation arc; 32 projections; 40 s/projection; 8 frames/heart cycle; and a 64 × 64 matrix. The studies were reconstructed using filtered back-projection without attenuation or scatter correction and realigned along the heart axis. A 17-segment model of polar map presentation was obtained from LV short-axis slices. Regions of low or absent ^99^mTc-tetrofosmin uptake indicated poor myocardial perfusion. Myocardial perfusion scintigraphy studies were categorized as normal (uniform uptake) or abnormal (global or regional defects).

### Non-isotopic cardiac status assessment

#### Troponin T

Troponin T levels were measured daily for seven days in 5 ml heparin plasma samples by electrochemiluminescence immunoassay with a COBAS™ C6000 analyzer (Roche Diagnostics, Basel, Switzerland). The reference range for upper normal limit was 0.03 μg/l, and the lower limit of detection was 0.01 μg/l.

#### Echocardiography

Transthoracic echocardiography (TTE) was performed during the first two days after admission with an ACUSON CV 70™ ultrasound system (Siemens Healthcare, Erlangen, Germany) equipped with a 2.5-MHz transducer. TTE was performed by one of two experienced cardiologists (FP and CJ) blinded to all clinical, hemodynamic, and biological data. Left ventricular ejection fraction (LVEF) was calculated by Simpson’s method. An LVEF more than 50% was defined as normal; an LVEF less than 50% was defined as reduced. Left ventricular filling pressure (LVFP) were assessed by E/A and E/Ea ratios.

### Neurological outcomes assessment

Neurological outcomes were assessed at one, three, and six months after SAH through a telephone interview of the patient or the functional rehabilitation practitioner using a modified Rankin Scale (mRS). This scale contains seven grades ranging from 0 (no symptoms at all) to 6 (death) [18]. For patients listed mRS 0 to mRS 3, quality of life was assessed at three and six months after SAH using the French version of the Medical Outcome Study Short Form-36 (SF-36) [19,20]. It is a generic health status measurement instrument composed of 36 questions and divided into two summarized scores: the 100-point physical component summary scale (PCS) and the 100-point mental component summary scale (MCS).

### Endpoints and sample size determination

The primary objective of this study was to evaluate cardiac glucose metabolism during the acute phase of SAH, including monthly follow-up of observed abnormalities. In a previous work, we had shown that a cardiac injury was present in 80% of patients [9]. We hypothesized that an abnormality of myocardial glucose metabolism would also be present in 80% of cases. A minimum of 28 study participants was necessary to obtain 15% precision around 80%, with a 95% confidence interval.

Secondary objectives were to assess the duration and reversibility of cardiac sympathetic impairment after SAH, and to compare cardiac and neurologic outcomes data according to the initial myocardial glucose metabolism status (normal/abnormal initial ^18^ F-FDG PET).

### Statistical analysis

Statistical analysis was performed with SPSS version 15.0 (SPSS Inc., Chicago, IL, USA), and data distributions were checked for normal distribution. Nominal variables are presented as numbers (%). Continuous variables are presented as the mean ± standard deviation (SD), or as the median [quartile 0.25 to quartile 0.75] when normal distribution was excluded. Comparison of two groups was performed using the Mann-Whitney *U* test and Fisher’s exact test. For all tests, *P* <0.05 was considered statistically significant.

## Results

### Patient characteristics

**Figure 1:**
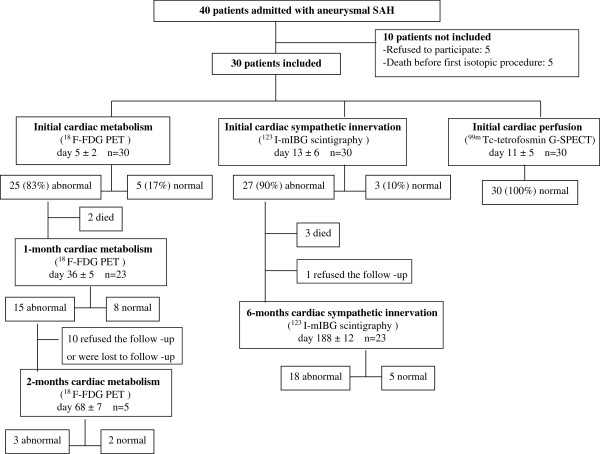
**Flow diagram of the study.** PET, positron emission tomography; G-SPECT, gated single-photon emission computed tomography.

During the study period, 40 patients were admitted to our ICU with acute-phase aneurysmal SAH. Five of patients refused to participate and five died before the first isotopic procedure. The remaining 30 patients were enrolled in the study (Figure 1). Patient characteristics, radiological data, and neurosurgical data are summarized in Table 1. No patient had evidence of prior coronary artery disease or diabetes.

**Table 1:** Population characteristics

**Age**, years *(mean ± SD)*	61 ± 12
**Female sex**, *n (%)*	22 (73%)
**BMI**, *kg/m*^*2*^*(mean ± SD)*	24.3 ± 2.7
**Past medical history**, *n (%):*	
*Active smoking*	14 (47%)
*Arterial hypertension*	9 (30%)
*Dyslipidemia*	4 (13%)
*Obesity (BMI >30 kg/m*^*2*^*)*	1 (3%)
*Diabetes mellitus*	0 (0%)
*Pheochromocytoma*	0 (0%)
*Thyroid disease (substituted hypothyroidism)*	1 (3%)
*Renal disease*	0 (0%)
*Liver disease*	0 (0%)
**Fisher grade ***(1/2/3/4), n (%)*	0 (0%)/3 (10%)/9 (30%)/18 (60%)
**WFNS score ***(1/2/3/4/5), n (%)*	12 (40%)/6 (20%)/3 (10%)/0 (0%)/9 (30%)
**Aneurysm position,** n (%)	
*ICA*	7 (24%)
*MCA*	10 (33%)
*AComA/ACA*	10 (33%)
*VA/BA*	1 (3%)
*PCA/PComA*	2 (7%)
**Aneurysm treatment**	
*Coiled, n (%)*	27 (90%)
*Craniotomy, n (%)*	3 (10%)
*Day of treatment, (mean ± SD)*	1.9 ± 0.7
**ICU period**, *n (%):*	
*Vasospasm*	14 (47%)
*Delayed cerebral ischemia*	8 (27%)
*Re-bleeding*	4 (13%)
*Hydrocephalus (derivated)*	16 (53%)
*Tracheostomy*	11 (37%)
**ICU length of stay**, *day (mean ± SD)*	16 ± 8
**Level of glucose just before **^**18**^**F-FDG PET**	139 ± 4
(*mg/dl, mean ± SD)*	

^18^F-FDG PET, ^18^F-fluorodesoxyglucose positron emission tomography; ACA, anterior cerebral artery; AcomA, anterior communicating artery; BA, basilar artery; BMI, body mass index; ICA, internal carotid artery; ICU, intensive care unit; MCA, middle cerebral artery; PCA, posterior cerebral artery; PComA, posterior communicating artery; SD, standard deviation; VA, vertebral artery; WFNS, World Federation of Neurosurgical Societies.

### Myocardial glucose metabolism

#### Initial ^18^F-FDG PET

All 30 patients first underwent PET on day 5 ± 2. Out of 30 patients, 25 patients (83%) revealed severely and diffusely reduced ^18^F-FDG LV uptake in a large area (mean defect of 54 ± 21%), where the defect pattern could not be explained by a single coronary artery distribution. Five patients (17%) exhibited normal ^18^F-FDG uptake (defect <15%), and their follow-up was stopped.

#### ^18^F-FDG PET follow-up

One month later, two additional patients had died, and PET was performed on day 36 ± 5 for 23 patients. ^18^F-FDG LV uptake was still impaired for 15 patients, with a mean defect area of 37 ± 17%. Eight patients exhibited normal ^18^F-FDG uptake, and their follow-up was stopped.

**Figure 2:**
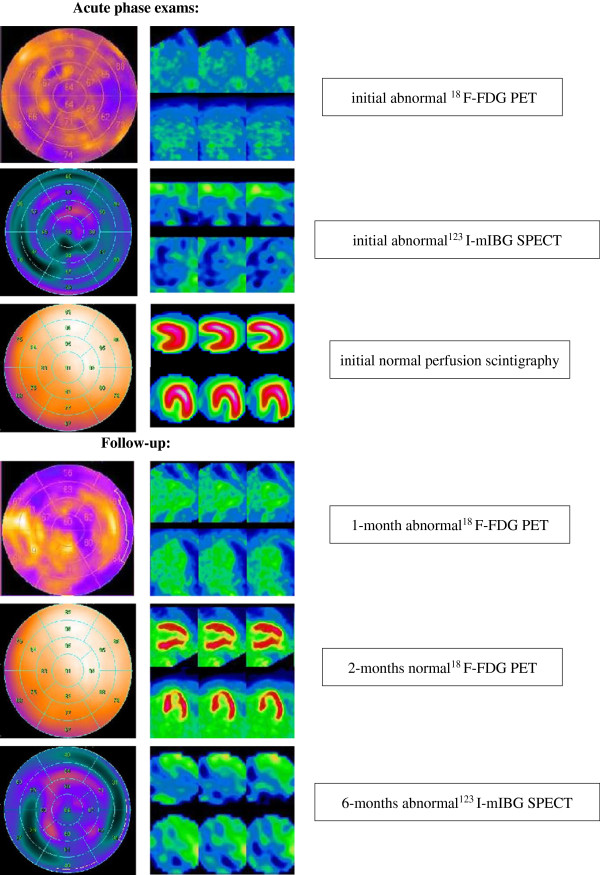
**Representative PET, SPECT, and scintigraphy findings.** Left ventricular transaxial slices (vertical long axis, horizontal long axis) and polar map presentation (17-segment model) of, respectively, cardiac ^18^F-FDG PET, ^123^I-mIBG SPECT, and perfusion scintigraphy performed during acute phase and follow-up in a patient with aneurysmal subarachnoid hemorrhage. We observed that the uptake of both ^18^F-FDG and ^123^I-mIBG were markedly reduced during the acute phase. The uptake of ^18^F-FDG was normalized two months later. The uptake of ^123^I-mIBG was still impaired six months after the onset of symptomatology. ^123^I-mIBG, ^123^I-meta-iodobenzylguanidine; ^18^F-FDG PET, ^18^F-fluorodesoxyglucose positron emission tomography; PET, positron emission tomography; SPECT, single-photon emission computed tomography.

Another one month later, PET was performed on day 68 ± 7 for only five patients. Indeed, 10 other patients refused the follow-up or were lost to follow-up at this time. ^18^F-FDG LV uptake was still impaired for three patients, with a mean defect area of 25 ± 6%. Two patients exhibited normal ^18^F-FDG uptake. The Figure 2 illustrated the exemplary case of a patient exhibiting normal ^18^F-FDG uptake two months after aneurysm rupture.

### Myocardial sympathetic innervation

#### Initial ^123^I-mIBG scintigraphy

All 30 patients underwent initial ^123^I-mIBG scintigraphy on day 13 ± 6. Twenty-seven patients (90%) exhibited reduced myocardial ^123^I-mIBG uptake (H/M ratio <1.75), and three patients (10%) exhibited normal uptake. For the 27 patients with reduced uptake, the mean H/M ratio was 1.38 ± 0.23. For the three patients with normal uptake, the mean H/M ratio was 1.87 ± 0.03. The mean LV ^123^I-mIBG uptake defect was, on average, 26 ± 18% in all patients, 28 ± 18% for the 27 patients with an abnormal H/M ratio, and 9 ± 7% for the three patients with a normal H/M ratio.

#### Sixth-month ^123^I-mIBG scintigraphy follow-up

Of the 27 patients with an abnormal initial ^123^I-mIBG scintigraphy, only 23 underwent a new examination six months later (day 188 ± 12). Indeed, three patients had died in the period since the previous examination, and one refused the follow-up exam. Of the 23 patients examined, five exhibited a normalized H/M ratio (1.92 ± 0.18); and 18 continued to have an abnormal H/M ratio (1.53 ± 0.22). For the 23 controlled patients, an H/M ratio mean global increase of 0.19 (13.7%) was noted between the initial examination and the six-month follow-up examination. After six months, the mean LV ^123^I-mIBG uptake defect was 17 ± 15%.

#### Myocardial perfusion

All 30 patients underwent initial cardiac gated ^99^mTc-tetrofosmin scintigraphy on day 11 ± 5. Myocardial perfusion of ^99^mTc-tetrofosmin was normal in all patients, and their follow-up was stopped.

### Non-isotopic cardiac damage assessment

The data are summarized in Table 2.

**Table 2:** **Initial gravity, cardiac damage, neurological outcomes, and quality-of-life assessment according to normal/abnormal initial **^**18**^ **F-FDG PET**

	**Initial normal PET (n = 5)**	**Initial abnormal PET (n = 25)**	**Initial normal PET vs. initial abnormal PET**
**Fisher grade ***(1/2/3/4), n (%)*	0 (0%)/0 (0%)/	0 (0%)/3 (12%)/	-
	1 (20%)/4 (80%)	8 (32%)/14 (56%)	
**WFNS ***(1/2/3/4/5), n (%)*	1 (20%)/1 (20%)/	11 (44%)/5 (20%)	-
	2 (40%)/0 (0%)/	/1 (4%)/0 (0%)/	
	1 (20%)	8 (32%)	
**Echocardiography ***(mean ± SD)*			
LVEF,%	64.2 ± 6.1	61.9 ± 4.6	NS
E/A	0.9 ± 0.3	1.1 ± 0.3	NS
E/Ea	6.1 ± 2.6	4.9 ± 2.4	NS
**Cardiac biomarkers ***[Median interquartile range]*			
Troponin T peak, μg/l	0.03 [0-0.04]	0 [0-0.09]	NS
**Neurological outcomes ***(mean ± SD)*			
mRS 1 month	4.5 ± 1.4	3.9 ± 1.4	NS
mRS 3 months	3.7 ± 1.8	3.2 ± 1.6	NS
mRS 6 months	3.3 ± 2.1	2.7 ± 1.8	NS
**Quality of life ***(mean ± SD)*			
MCS 3 months	63 ± 6	46 ± 18	NS
MCS 6 months	54 ± 11	55 ± 17	NS
PCS 3 months	64 ± 7	44 ± 15	NS
PCS 6 months	57 ± 21	55 ± 19	NS

Troponin T values denote the peak daily dosage during the first seven days. Statistical significance was accepted at *P* <0.05. ^18^ F-FDG PET, ^18^ F-fluorodesoxyglucose positron emission tomography; LVEF, left ventricular ejection fraction; mRS, modified Rankin Scale; MCS, mental component summary scale; NS, non-significant; PCS, physical component summary scale; WFNS, World Federation of Neurosurgical Societies.

All 30 included patients underwent TTE during the first two days after admission. All of them exhibited normal LV systolic function, with a mean LVEF (assessed by Simpson’s method) of 62.3 ± 4.9%. Regarding LV diastolic parameters, filling pressures were low with a mean E/A ratio of 1.0 ± 0.3, and a mean E/Ea ratio of 5.1 ± 2.5.

#### Troponin T

During the first seven days, an abnormal troponin T level increase (>0.03 μg/l) was noted for 11 patients (37%), with a median peak level of 0.15 [0.07 to 0.36] μg/l, and occurred on average on day 2 ± 1.8. This group of 11 patients with abnormal troponin T levels was characterized by a mean WFNS score of 3.5 ± 1.8, a mean Fisher grade of 3.7 ± 0.5, and mRS scores of 4.5 ± 1.0 (one-month follow-up) and 3.0 ± 1.8 (six-month follow-up). In comparison with the group of 19 patients with normal troponin T levels, no significantly differences were observed with regard to initial gravity or neurological outcome.

### Neurological outcomes and quality of life assessment

**Figure 3:**
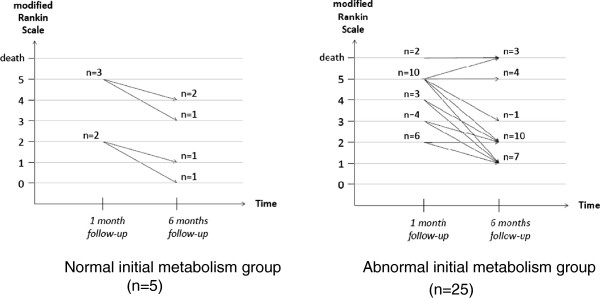
Modified Rankin Scale score distribution in both groups at one and six months of follow-up.

Neurological outcomes assessed by mRS are summarized in Table 2 and Figure 3. The mean mRS scores were 3.9 ± 1.4 after one month, 3.2 ± 1.6 after three months, and 2.7 ± 1.8 after six months. Regarding quality of life assessed by SF-36 (Table 2), the mean PCS was 47 ± 16 after three months and 56 ± 20 after six months, and the mean MCS was 49 ± 18 after three months and 55 ± 16 after six months.

Vasospasm concerned 14 patients and delayed cerebral ischemia concerned eight patients. This group of eight patients with delayed cerebral ischemia was characterized by a mean WFNS score of 3.8 ± 1.8, a mean Fisher grade of 3.9 ± 0.4, abnormal initial ^18^F-FDG uptake in 75%, abnormal initial ^123^I-mIBG uptake in 100%, abnormal troponin T levels in 62.5%, a mean LVEF of 65 ± 6%, and modified Rankin Scale scores of 4.9 ± 0.8 (one-month follow-up) and 4.1 ± 1.4 (six-month follow-up).

### Initial gravity and outcomes data according to normal/abnormal initial ^18^F-FDG PET

Initial gravity (Fisher grade and WFNS), cardiac damage, neurological outcomes, and quality-of-life assessments of the five patients with normal initial cardiac glucose metabolism were compared with those from the 25 patients who initially exhibited abnormal cardiac glucose metabolism (Table 2). No significantly differences were observed with regard to Fisher grade, WFNS, systolic or diastolic echocardiographic parameters; troponin T level; neurological outcomes with mRS scores at one, three, and six months (Figure 3); or quality of life (SF-36, MCS and PCS) at three and six months. Acute phase altered cardiac glucose metabolism was not associated with more unfavorable cardiac or neurological outcomes.

## Discussion

### Impairment of cardiac glucose metabolism

To the best of our knowledge, this is the first study to focus on disturbances in cardiac glucose metabolism after SAH. The obtained results established the initial existence of a major impairment of cardiac glucose metabolism, with a LV ^18^F-FDG uptake severely and globally affected. The defect distribution was heterogeneous and concerned the LV in a non-systematized way. In the same time, cardiac perfusion was not impaired.

Impairment of cardiac metabolism has been revealed in various situations of sympathetic stimulation such as chronic heart failure [21] and Takotsubo cardiomyopathy (TTC) [22-25]. Chronic heart failure causes a state of chronic exaggerated sympathetic stimulation in which cardiac glucose metabolism is impaired. Taylor *et al*. demonstrated in 2001 that cardiac ^18^F-FDG uptake was lower in heart failure patients than in healthy volunteers [21]. TTC, also known as transient LV apical ballooning syndrome, is another neurogenic stress cardiomyopathy that causes transient LV dysfunction in patients under emotional or physical stress [26-28]. The acute stress cardiomyopathy after SAH has often been compared with TTC [10,12,29]. TTC has been well investigated by cardiac nuclear medical techniques [22-25,30]. ^18^F-FDG PET assessment of myocardial glucose metabolism shows severe impairment, with ^18^F-FDG uptake reduced among 87 to 100% of TTC patients during the acute phase [22-24]. The areas affected by this defect were the apical and midventricular segments [23,24]. The mean extent of the ^18^F-FDG uptake defect was 33 ± 15% [23]. Furthermore, ^18^F-FDG PET studies revealed a strong correlation between myocardial metabolism defects and the location of wall motion abnormality on TTE [23]. At the same time, myocardial perfusion scintigraphy was normal in all patients [23]. Follow-up assessments depicted the normalization of ^18^F-FDG uptake at three months in all patients [24].

Finally, the initial impairment of cardiac glucose metabolism after SAH (83% of patients, mean defect 54 ± 21%) was more diffuse than that of TTC (87 to 100% of patients, mean defect 33 ± 15%). The duration of reversibility of the ^18^F-FDG uptake defects appeared to be almost similar.

### Impairment of cardiac sympathetic innervation

According to our results, the impairment of cardiac sympathetic innervation during the acute stage of SAH affected a large majority of patients. Myocardial ^123^I-mIBG uptake was severely and globally affected. Its distribution was heterogeneous and affected the LV in a non-systematized way. This infringement was slowly reversible; the six-month follow-up revealed that the condition persisted in the majority of affected patients.

The scientific literature concerning isotopic exploration of myocardial sympathetic innervation after SAH is poor, with only a single human study [11]. In this trial, 41 patients underwent myocardial ^123^I-mIBG and perfusion scintigraphy during the acute stage of SAH. ^123^I-mIBG uptake was abnormal in 12 patients (29%), with nine global defects and three regional defects. However, the used ^123^I-mIBG scintigraphic protocol was different to ours. Indeed, acquisition was performed only 15 minutes after radiotracer injection. Now, it has been established that norepinephrine and ^123^I-mIBG shared an active neuronal recapture mechanism and a passive extraneuronal mechanism. ^123^I-mIBG uptake was mainly extraneuronal at 5 minutes and neuronal at 3 h [31,32]. ^123^I-mIBG scintigraphy performed on denervated dogs at 5 minutes and at 3 h showed that the ^123^I-mIBG uptake was normal at 5 minutes but deeply reduced at 3 h [31,32]. Considering the washout of extraneuronal ^123^I-mIBG, late cardiac ^123^I-mIBG uptake (at 3 h) better reflects neuronal ^123^I-mIBG uptake. Moreover, this late cardiac ^123^I-mIBG uptake is correlated with the myocardial norepinephrine concentration [33].

In TTC patients, ^123^I-mIBG scintigraphy revealed altered cardiac sympathetic innervation, with absent or strongly reduced tracer uptake at the hypocontractile zones (mean LV defect, 38 ± 17%) [23,30]. The topography and extent of glucose metabolism defects (^18^ F-FDG) and sympathetic innervation abnormalities (^123^I-mIBG) were largely overlapping [23]. At 12 months and despite progressive evolution, all controlled patients presented with incomplete recovery of apical ^123^I-mIBG uptake [23]. Finally, the initial impairment of cardiac sympathetic innervation after SAH (90% of patients, mean defect 28 ± 18%) differed from that of TTC (100% of patients, mean defect 38 ± 17%) regarding its heterogeneous and non-systematized distribution.

### Neurogenic stunned myocardium

A link between morbidity and mortality after SAH and concomitant cardiac complications is now well established [6,34,35]. Recently, van der Bilt *et al*. studied the relationship between cardiac dysfunction after aneurysmal SAH and neurological outcome. They established in particular that wall motion abnormalities on TTE are independent risk factors for clinical outcome, partly explained by a higher risk of delayed cerebral ischemia [35]. Coronary angiography [36] and perfusion scintigraphy [11] have demonstrated that the myocardial damage does not result from ischemia. The most widely accepted theory for SAH-induced neurogenic myocardial stunning is the ‘catecholamine hypothesis’. The release of massive quantities of catecholamines following aneurysm rupture results in specific myocardial lesions [37].

The transient regional metabolic disorder is considered to be the metabolic state of stunned myocardium [23]. Catecholamine-mediated myocardial insulin resistance may be responsible for reduced ^18^F-FDG uptake in the hypocontractile regions [38]. The inhibition of intracellular translocation of glucose transporters (GLUT-4) by calcium overload may also contribute to the reduced ^18^F-FDG uptake in cardiocytes [39]. The concordance of ^123^I-mIBG and ^18^F-FDG uptake abnormalities, as well as their common temporal evolution, emphasize the close relationship between myocardial sympathetic function and glucose metabolism.

Our results established that neither cardiac glucose metabolism nor sympathetic innervation impairment resulted in major LV systolic or diastolic dysfunction in these 30 patients. Although similar results were previously described [9], others studies showed LV systolic dysfunction in 22 to 38% acute phase SAH patients [6,40]. This fact probably resulted from our exclusion criteria of patients with a major hemodynamic instability preventing intrahospital transport to the Nuclear Medicine Department. Conversely to our SAH patients, TTC causes LV dysfunction, with hypocontractile segments characterized by normal perfusion but reduced uptake of ^18^F-FDG and ^123^I-mIBG. These data likely attest to different pathophysiological mechanisms underlying TTC due to stressful events and SAH-related cardiopathy due to aneurysm rupture and acute intracranial hypertension.

Nevertheless, the numerous similarities between SAH-related myocardiopathy and TTC allow many authors to believe that these two entities form part of a single nosologic group of ‘neurogenic stress cardiomyopathy’, also termed ‘neurogenic stunned myocardium’ [10,12,28,29].

### Study limitations

First, the study design was based on a single-center prospective recruitment with small numbers. Second, although the study population was representative of real-life SAH, external validity of the study was reduced because of case selection bias. Indeed, patients dead before the first isotopic exam were excluded, but these represented the gravest cases, often with major hemodynamic instability, and their exclusion constituted a loss of relevant information regarding acute neurogenic stress cardiomyopathy. Third, myocardial perfusion scintigraphy was performed late in the course of SAH (day 11 ± 5). Earlier assessment of myocardial perfusion might have provided further information on the potential role of myocardial ischemia-vasospasm on the pathogenesis of potential alterations in cardiac metabolism and contractility.

## Conclusions

This preliminary study contributes modestly to progress in the knowledge of neurogenic heart disorder after SAH. In hemodynamically stable acute phase SAH patients, cardiomyopathy is characterized by diffuse and heterogeneous ^18^F-FDG and ^123^I-mIBG uptake defect, contrasting with an absence of significant functional consequences to LV systolic function and segmental kinetics. Additional research is necessary to increase pathophysiological understanding of these diseases.

## Key messages

• In hemodynamically stable acute phase SAH patients, cardiomyopathy is characterized by an impairment of cardiac metabolism (diffuse and heterogeneous ^18^F-FDG uptake defect).

• In hemodynamically stable acute phase SAH patients, cardiomyopathy is characterized by an impairment of sympathetic innervation (diffuse and heterogeneous ^123^I-mIBG uptake defect).

## Abbreviations

^123^I-mIBG: ^123^I-meta-iodobenzylguanidine; ^18^ F-FDG PET: ^18^ F-fluorodesoxyglucose positron emission tomography; BNP: B-type natriuretic peptide; CT: computed tomography; H/M: heart to mediastinal; (G-)SPECT: (gated) single-photon emission computed tomography; LV: left ventricle; LVEF: left ventricular ejection fraction; LVFP: left ventricular filling pressure; MCS: mental component summary scale; mRS: modified Rankin Scale; PCS: physical component summary scale; ROI: region of interest; SAH: subarachnoid hemorrhage; SF-36: 36-item short form health survey; TTC: Takotsubo cardiomyopathy; TTE: transthoracic echocardiography; WFNS: World Federation of Neurosurgical Societies.

## Competing interests

The authors declare that they have no competing interests.

## Authors’ contributions

BP contributed to the study concept and design, acquisition of data, analysis and interpretation of data, the drafting of the manuscript and critically revising the manuscript for important intellectual content. MB contributed to the study concept and design, acquisition of data, analysis and interpretation of data, the drafting of the manuscript and critically revising the manuscript for important intellectual content. ED contributed to the study concept and design and acquisition of data. PC contributed to the study concept and design, acquisition of data, analysis and interpretation of data and the drafting of the manuscript. FP contributed to the study concept and design and acquisition of data. SC contributed to the study concept and design, analysis and interpretation of data and critically revising the manuscript for important intellectual content. CJ contributed to the acquisition of data and the drafting of the manuscript. EB contributed to the acquisition of data, analysis and interpretation of data and the drafting of the manuscript. EM contributed to the acquisition of data, analysis and interpretation of data, the drafting of the manuscript and critically revising the manuscript for important intellectual content. AD contributed to the analysis and interpretation of data, the drafting of the manuscript and critically revising the manuscript for important intellectual content. ND contributed to the analysis and interpretation of data and critically revising the manuscript for important intellectual content. JBV contributed to the analysis and interpretation of data. GL contributed to the analysis and interpretation of data and critically revising the manuscript for important intellectual content. PE contributed to the analysis and interpretation of data and critically revising the manuscript for important intellectual content. HB contributed to the analysis and interpretation of data and the drafting of the manuscript. PG contributed to the analysis and interpretation of data and the drafting of the manuscript. GH contributed to the analysis and interpretation of data and critically revising the manuscript for important intellectual content. All authors read and approved the final manuscript.
